# Toxidromes for Working Dogs

**DOI:** 10.3389/fvets.2022.898100

**Published:** 2022-07-15

**Authors:** Maureen A. McMichael, Melissa Singletary, Benson T. Akingbemi

**Affiliations:** ^1^Department of Clinical Sciences, College of Veterinary Medicine, Auburn University, Auburn, AL, United States; ^2^Department of Anatomy, Physiology and Pharmacology, College of Veterinary Medicine, Auburn University, Auburn, AL, United States; ^3^Canine Performance Sciences, College of Veterinary Medicine, Auburn University, Auburn, AL, United States

**Keywords:** K9, canine, opioid, search and rescue, military working dog, naloxone, Narcan®, organophosphate

## Abstract

Terrorist attacks with biological and chemical warfare agents are increasing in frequency worldwide. Additionally, hazardous chemical accidents, illicit drug laboratories and intentional poisonings are potential sites for exposure to working dogs. Working dogs play a crucial role in law enforcement, military and search and rescue teams. Their intelligence, agility and strength make them ideal partners to be deployed to these natural disaster sites, terrorist attacks and industrial accidents. This, unfortunately, leads to increasing exposure to chemical and biological weapons and other hazardous substances. First responders have little to no training in emergency care of working dogs and veterinarians have very little training on recognition of the clinical signs of many of these agents. In order to ensure a rapid medical response at the scene first responders and veterinarians need a primer on these agents. Identifying a specific agent amidst the chaos of a mass casualty event is challenging. Toxidromes are a constellation of clinical and/or laboratory findings that allow for rapid identification of the clinical signs associated with a class of toxin and have been helpful in human medical triage. Focusing on a class of agents rather than on each individual toxin, allows for more expedient administration of antidotes and appropriate supportive care. This article reviews toxidromes for the most common chemical weapons with a special emphasis on clinical signs that are specific (and different) for canines as well as appropriate antidotes for working canines. To our knowledge, there are no publications describing toxidromes for working dogs.

## Introduction

Toxic syndromes, called toxidromes, are a group of clinical signs that are characteristic of a specific class of toxin. Focusing on a class of agents rather than on each individual toxin allows for rapid identification of common clinical signs associated with that particular class of toxin. Ideally an antidote or treatment will be identified and administered more expediently using this identification scheme. Toxidromes are not new to human medicine and recently they have been adapted for human medical triage concerning chemical weapons attacks (1, 2). Most toxidrome schemes are designed for first responders, emergency medical service (EMS) personnel or ER physicians, who may arrive on the scene of a possible terrorist attack and need to intervene emergently. Toxidromes are most useful when the rapid identification of a life threatening toxicosis is needed, particularly at chaotic scenes with mass casualties. Toxidromes can encompass more than physical examination findings to include point of care and laboratory testing, ECG observations, or may even rely on just one clinical sign (e.g., hyperthermic toxidromes).

As more working dogs are deployed worldwide to natural disaster sites, terrorist attacks, and industrial accidents, it is likely that these dogs (e.g., search and rescue, law enforcement canines, military working dogs, etc.) will be present at these scenes. These highly trained and extremely valuable canines are at equal or greater risk of exposure to agents used in these attacks. It is imperative that first responders and veterinarians be familiar with the clinical signs of these agent classes in canines, particularly in the ways they may differ from clinical signs in humans (3). It is also essential to know the appropriate treatment (e.g., antidote, supportive care, etc.) as well as correct dosages to reverse or mitigate toxicosis in canines. Various forms of toxicants (e.g., liquid, solid, particulate, etc.) as well as their most likely routes of exposure have been covered elsewhere (3). There are a plethora of potential toxicologic agent exposures possible and those not discussed here have been covered elsewhere (4–6).

This article reviews toxidromes for the most common chemical weapons with a special emphasis on clinical signs and antidote dosages for canines. We have included a separate section on personal protective equipment (PPE) and decontamination (decon) for the toxidromes described. To our knowledge, there are no publications describing toxidromes for working canines.

## Toxidromes for Human Medicine

Canines differ from humans in several important ways. Humans maintain a bipedal upright position, while their canine companion is considerably lower to the ground as a quadruped. This predisposes the canine to contact and inhalation exposures with heavier substances or powders that may be present at ground level. The risk is increased as a significant portion of working dogs are detection based and classically work in a nose-down tail-up manner, making inhalation of a potential toxin more likely.

There are important differences to highlight between human and canine clinical signs (e.g., sweat glands in dogs are limited to their paws so they won't show profuse sweating—a classic distinguishing feature of the cholinergic or nerve agent toxidrome), as well as differences in dosage and response to antidotes which are critical to proper diagnosis, management, care and treatment. Working dogs may have a higher body temperature during work than pet dogs and some military working dogs reach temperatures of 41.1°C (106°F) routinely (7). Importantly, canine specific equipment may be required for some management procedures. For example, oxygen masks used for humans don't fit over the typical working dog's long snout. Endotracheal (ET) tubes are generally larger than those for humans of the same weight with a typically sized working dog (25–35 kg) requiring a size 8 or 9 ET tube (Table 1).

**Table 1:** K9 specific equipment and physical parameters.

**Procedure/Parameter**	**Canine**	**Resources**
Oxygen delivery (Mask)	Cone shaped to fit snout, size 5	Reference #13 (Mitek et al.)
Endotracheal tube size	20 kg dog = size 10 mm (if 9 mm is the largest available it should be used). 6–8 kg dog = 7 mm, 10–12 kg dog = 8 mm, 14–16 kg dog = 9 mm, 18–20 kg dog = 10 mm, 25 kg = 11 mm, 30 kg dog = 12 mm.	Reference #13 (Mitek et al.)
Intubation	Relatively easy to visualize arytenoids	Reference #13 (Mitek et al.)
Heart rate	60–100 bpm, athletic K9s may go to 40 bpm	Reference #3 (Palmer L)
Respiratory rate	10–12 bpm at rest	Reference #3 (Palmer L)
Temperature at rest	37.8–38.3°C (100–101°F)	Reference #3 (Palmer L)
Temperature during, after working	Up to 40.6°C (105°F) and higher	Reference #3 (Palmer L)

Therefore, veterinarians, canine handlers, and all first responders exposed to working canines need resources for rapid identification and treatment of the most common chemical agents that are most likely to be encountered.

**Figure 1:**
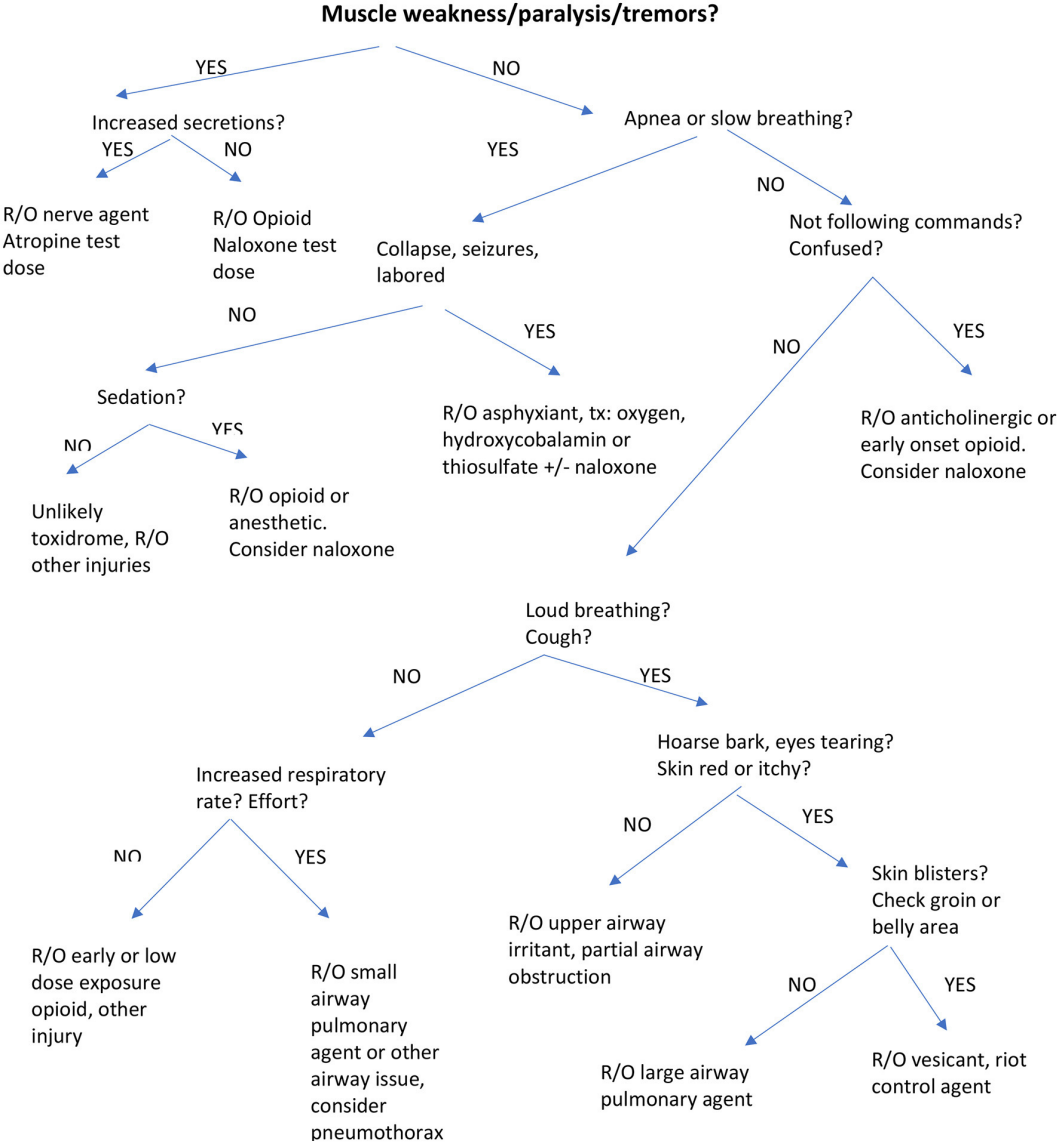
Algorithm for identification of Toxidromes muscle weakness/paralysis/tremors or increased secretions?

The canine toxidromes reviewed here resemble, in part, human toxidromes recently published (1, 2). We have ordered these in terms of clinical onset (the most rapid onset agents) and lethality (the most lethal agents) and availability of an antidote collectively weighted first in order of discussion. The last toxidromes are those that are less likely to be lethal and have no antidote, limiting treatment only to supportive care. The toxidrome descriptions are followed by a table with clinical signs, treatment and antidote dosages for canines (Table 2). We have also included an algorithm for veterinarians and first responders to enable rapid identification of a specific toxidrome (Figure 1).

**Table 2:** Emergency treatment.

**Toxicant Class**	**Toxidrome Signs**	Emergency Treatment^*^	**Antidotes**
Opioids/opiates	Mild: drowsiness, ataxia, sedation Severe: collapse, apnea Miosis may not occur	Secure airway, provide manual ventilation if not ventilating^*^	Naloxone (Narcan®) at 4 mg per K9 IN, IM, IV. Repeat q10 min until effect (mentation improves) (8).
Nerve Agents Organophosphates Carbamates	Salivation, lacrimation, urination, defecation, and emesis. May see bradycardia, bronchospasm, and bradypnea. May see tachycardia, miosis or mydriasis. Muscle twitching, seizures, paralysis, and apnea may occur.	Secure airway, provide manual ventilation if not ventilating^*^ Autoinjectors provide 2 mg atropine and 600 mg 2-PAM. Both DuoDote® and ATNA are single injection, while Mark I Kits are 2 separate autoinjectors in one kit. These dosages are ok for 25 kg K9. Repeat q10–15min until respiratory secretions dry.	Atropine test dose @ 0.02 mg/kg (0.5 mg per 25 kg K9). If K9 responds (eyes dilate, saliva stops, HR increases) unlikely OP. D/C atropine (9). If no response give atropine @ 0.1 mg/kg (2.5 mg per 25 kg K9) at $\frac{1}{4}$ dose IV, remainder IM and 2-PAM @ 20 mg/kg (500 mg per 25 kg K9) IM or very slow IV until muscle weakness improves (10, 11). Midazolam IM,IV @ 0.2–0.4 mg/kg or diazepam IV @2–5 mg/kg (12, 13).
4th Generation Nerve Agents	Above plus bronchoconstriction, seizures	Secure airway, provide manual ventilation if not ventilating	Treat as for nerve agents (above). If severe clinical signs consider IV Lipids with 20% lipids @1.5 mL/kg over 15 min, then 0.25 mL/kg/min × 30–60 min (14).
Asphyxiants Knockdown Agents	Mild: restless, fatigue, tachycardia, tachypnea, vomiting Mod-severe: seizures, bradycardia, apnea, CPA CO: hypotension, cherry red mm H_2_S: rotten egg odor, corneal ulcerations Cyanide: almond smell, cherry red skin	Remove to fresh air, oxygen via K9 mask or secure airway, provide manual ventilation if not ventilating	Hydroxycobalamin @ 150 mg/kg or 3.75 grams per 25 kg K9 given IV over 7.5 min (15).
Solvents Anesthetics Sedatives	Sedation, ataxia, stupor, coma, cardiac arrest	Remove to fresh air, oxygen via K9 mask or secure airway, provide manual ventilation if not ventilating	Naloxone (Narcan®) at 4 mg per K9 IN, IM, IV. Repeat q10 min until effect (mentation improves) (8).
Primary Respiratory Agents Upper Airway	Loud respiratory noise, cough, increased respiratory rate and effort, upper airway obstruction	Remove to fresh air, flush eyes, nose, mouth, oxygen via face mask	Albuterol inhaler (1 puff), consider anti-inflammatory dose of glucocorticoids if severe, nebulization of sodium bicarbonate for chlorine gas inhalation (16).
Primary Respiratory Agents Lower Airway	Increased respiratory rate and effort, cough	Remove to fresh air, flush eyes, nose, mouth, oxygen via face mask	Albuterol inhaler (1 puff), consider anti-inflammatory dose of glucocorticoids if severe, N-acetylcysteine @ 70 mg/kg IV over 30 min, diluted 1:2 (17).
Vesicants Riot Control T2 Toxins	Ocular irritation (ulcers, redness, lacrimation), skin blisters (esp. inguinal and axillary areas)	Remove to fresh air, flush eyes, nose, mouth, oxygen via face mask	Ophthalmic ointments, e-collar to prevent scratching eyes.
Anticholinergics	Tachycardia, hypertension, mental dullness, delirium, mydriasis, hyperthermia	Cool down with cold (not freezing) water, fans, IV fluids	For seizures, midazolam IM, IV @ 0.2–0.4 mg/kg or diazepam IV @2–5 mg/kg (12, 13). Physostigmine @ 0.02 mg/kg IV or neostigmine at 1–2 mg IM (18, 19).

*CO, Carbon monoxide*.

*CPA, Cardiopulmonary arrest*.

* *ET intubation or tight fitting 02 mask attached to AMBU-bag® or BVM. Ventilate at 12–15 breaths per minute. If CPR is needed do NOT perform mouth to snout ventilation due to potential for toxicant exposure*.

## Personal Protective Equipment and Decontamination

Personal protective equipment (PPE) is essential to protect the first responder as well as to prevent cross contamination. All toxidromes listed here require PPE. Decontamination (Decon) is essential for the potential exposures listed here as well as for unknown exposures. There is some debate as to optimal decontamination of the working K9. A study comparing standard FEMA K9 decontamination with a new protocol using soft rubber grooming brushes, a specific body wash and a grated floor found the new protocol to be superior in removing an oil based “toxin” from canines (20). First responders should be familiar with current standard decontamination protocols for canines. A thorough overview of evacuation and decontamination for dogs exposed during mass exposures has been published (21). Standard decontamination will be discussed here, toxidrome specific decontamination, where applicable, will be listed under each toxidrome.

Both the canine and the tack (e.g., collar, vest, harness, etc.) need to be decontaminated after potential exposures. The safest method of canine decontamination is to start with a 10 s dry blot (e.g., blot the canine's skin using a dry paper towel, paying particular attention to the hairless areas (e.g., groin, under front limbs). This can be followed by a 10 s dry rub followed by a high volume, low pressure bath with soap, followed by gentle rubbing with dry towels. For powder or fine particulate matter contamination, alternative options include using damp disposable towels to wipe down the canine coat followed by high volume, low pressure flushing of the hair with liquid soap and water. If a washing station is available the eyes and mouth can be washed out as well. Alternatively, the eyes can be washed out using contact lens solution or other balanced salt solution. It is essential that the head be held down while washing out the mouth to avoid getting water in the airways.

## Emesis

Emesis is a vital component of the treatment of several types of toxin ingestion. Since many of the potential ingestions covered in this article will likely be unknown or unclear (e.g., mixed toxin exposure) and emesis may be harmful in many conditions, we have elected not to include it under each toxidrome. Emesis is contraindicated with mental depression, seizures, neurologic signs, or with toxins likely to cause these. It is also contraindicated with caustic or corrosive substances or with petroleum distillates, oil or gasoline ingestions. When emesis is considered it is best done early, ideally <1 h after ingestion as recovery of the toxin decreases over time. If the canine is a good candidate for emesis, apomorphine can be used at 0.02 to 0.04 mg/kg IV or IM or via the conjunctival sac. When administered via the conjunctival sac the pill is mixed with sterile water to prevent irritation of the eye. Vomiting usually occurs within 4–6 min and the eye can be flushed out if that route was used. Alternatively, hydrogen peroxide given orally at 2.2 mL/kg for a maximum of 45 mL per dog can be attempted with most dogs vomiting within 15 min (22). Ropinirole ophthalmic solution appears to be safe, effective and is easily administered in the eye (23). For dogs weighing 20–35 kg 2 drops in each eye was an effective emetic in 97% of dogs in one study (23).

### Opioids/Opiates

#### Overview

Opioids include naturally derived substances from the Poppy plant (opiates) as well as synthetic substances produced in laboratories. Examples of opiates are opium, codeine and morphine and examples of synthetics (and semi-synthetics) include fentanyl, carfentanil, sufentanil, methadone, oxycodone, hydrocodone, and hydromorphone. Newer synthetics include U47700, MT-45, and precursor agents (4-aminophenyl-1-phenylethylpiperidine [4-ANPP] and N-phenyl-1-{2-phenylehtyly}−4-piperidinamine [NPP]). The precursor agents are intermediates in the synthesis of fentanyl and require additional steps/agents to complete the synthesis of the final product. The various opioids have different affinities for the mu (OP_3_), kappa (OP_2_), and delta (OP_1_) receptors. Due to the worldwide opioid crisis working dogs are being exposed to potentially toxic levels of opioids (24). Several of the synthetics are highly potent, for example, fentanyl is ~100 times more potent than morphine and carfentanil is approximately 10,000 times more potent than morphine (25, 26). Exposure to a quantity of carfentanil powder the size of a poppy seed is believed to be fatal and this can occur via mucous membrane exposure (e.g., eyes, mouth, nose) or inhalation.

Some opioids are known to cause seizures (e.g., tramadol and meperidine) and cardiac QT interval prolongation (e.g., methadone) (27). Wooden chest syndrome was also reported with fentanyl exposure in humans which causes severe rigidity of the thoracic cage making ventilation (either negative or positive pressure ventilation) difficult (28). In a study of Beagle dogs on equipotent doses of isoflurane, given either a low dose (33 mcg/kg loading followed by 0.2 mcg/kg/min) or a high dose (102 mcg/kg loading followed by 0.8 mcg/kg/min) of fentanyl reported no chest wall rigidity with either dose (29). However, it is not clear that the signs which occur in humans also occur in canines to an appreciable extent.

#### Clinical Signs

Opioid poisoning can exert a variety of clinical signs depending on the specific opioid, the potency, and if any other agents are present (30). It is becoming more common for cocaine, heroin, and even marijuana to be mixed with potent opioids leading to difficulties in the interpretation of the clinical syndrome. A classic, pure opioid syndrome includes respiratory depression, mental depression and miosis.

In a 2018 study of fentanyl in healthy working dogs, researchers reported clinical signs ranging from mild drowsiness and ataxia to complete sedation (8). Interestingly, the dogs responded differently to the same dosage of fentanyl that was adjusted for weight. After intranasal naloxone administration, there were also a variety of responses (e.g., slow recovery to rapid reversal and hyperactivity) (8). The varying nature of clinical signs and response to treatment highlights the fact that opioid exposure should be considered for a wide range of clinical signs and naloxone should be given to effect.

#### Treatment

##### Standard Decon and PPE Required

Treatment of opioid toxicosis consists of decontamination, administration of naloxone, and if needed, securing the airway and manually ventilating for the canine while on route to a veterinary emergency facility.

The powdered form of opioids may become aerosolized, causing contamination of the canine's coat. Opioids are pulverized into a fine particulate matter in clandestine tableting facilities and extra caution is suggested when entering these buildings. Decontamination of the canine should occur after naloxone has been administered.

Naloxone (Narcan®) can be given by the IV, IM, or intranasal (IN) routes. The IN route is convenient but may be difficult in a dog with a basket muzzle on. The IM route is a good next choice and naloxone is given in the front of the thigh angling toward the head. Alternatively, the epaxial muscles on either side of the spine are good options. The IV route, although the most direct, should only be used if intravenous access is already in place. The time it takes to gain IV access may compromise the patient's safety. Naloxone administration is repeated every 2–5 min until effect is achieved. See Table 2 for dosages.

Securing the airway in a K9 in the field can be challenging even for trained professionals with appropriate equipment. Options include endotracheal tube (ET) intubation or secure face mask ventilation and will depend on the training level of the first responder as well as equipment available (31). Intubating a canine is generally easier than intubating a human and is a skill that EMS professionals can be taught quickly. Endotracheal Tube sizes are listed in Table 1. Once the canine is intubated and the ET tube secured it can be attached to a bag-valve-mask (BVM) or an AMBU® bag for ventilation at 12–15 breaths per minute. Secure face mask ventilation is a quick, simple option that can be attempted in non-breathing canines when ET intubation is not possible. This involves placing a canine oxygen facemask (sizes provided in Table 1) to cover the snout (e.g., nose and mouth) and securing it so that air will not leak out through the wide end of the cone. This can be achieved by stuffing gauze around the opening. The oxygen mask is then attached to either the BVM or the AMBU® bag with ventilation provided at 12–15 breaths per minute. There is a risk of forcing air into the stomach so caution is recommended. The chest should rise during positive pressure ventilation and the abdomen should not rise and should not become distended. If the canine is still unconscious, naloxone treatment is repeated and the canine is then transported to the nearest veterinary emergency facility.

^**^Mouth to Snout Ventilation should not be attempted due to potential risk for rescuer exposure to opioids. Endotracheal intubation should never be attempted in a conscious animal^**^.

*Clinical Pearl: Working dogs are stoic and may not show clinical signs until they collapse. It is essential to get naloxone into them as soon as possible. If they are not ventilating (the chest is not moving) they will need ventilation until the naloxone reverses the opioid*.

### Nerve Agents/Cholinergics/Pesticides

#### Overview

Exposure to cholinergic agents results in accumulation of acetylcholine (ACh) due to inhibition of the enzyme (acetylcholinesterase; AChE) that normally breaks it down. ACh activates/excites many tissues in the body but when it is uninhibited, what starts out as excitation (e.g., muscle tremors and stiffness) can lead to unresponsive tissues (e.g., paralysis) (9, 32). This toxidrome leads to four categories of clinical signs; muscarinic, nicotinic, CNS, and intermediate. The clinical signs are thought to occur in this order; muscarinic signs first, followed by nicotinic, with CNS signs last. In clinical cases, it may not be possible to differentiate these clearly (33). See clinical signs below for details.

Death is usually associated with respiratory failure due to excess respiratory secretions, CNS driven respiratory depression, paralysis of the diaphragm, and bronchoconstriction (9).

The toxin may be absorbed via skin, GI tract, mucous membrane (e.g., eyes, mouth, nose) or through respiratory exposure. Compounds in this class include organophosphates (chlorpyrifos, parathion, malathion, diazinon, etc.), carbamates (carbofuran, carbaryl, etc.), and nerve agents (sarin, VX, Novichoks).

The fourth-generation nerve agents (FGAs; also called Novichoks) persist on the skin and in the environment for days and are highly fatal. These may have a latency period with clinical signs manifesting hours to days after exposure. More information on FGAs is at the end of section Nerve Agents/Cholinergics/Pesticides.

#### Clinical Signs

Clinical signs vary because several cholinergic toxins may activate the sympathetic nervous system (e.g., organophosphates) making clinical signs confusing.

#### Muscarinic Signs

Overstimulation of muscarinic receptors results in parasympathetic signs of excess secretions (Salivation, Lacrimation, Urination, Defecation, Gastric signs, Emesis; SLUDGE) and Bradycardia, Bronchospasm, and Bronchorrhea (triple B). Miosis may be seen if there is not an overstimulation of the sympathetic nervous system causing mydriasis. Coughing due to respiratory secretions can be prominent. As mentioned, profuse sweating is a classic sign seen in humans but canines have few sweat glands with merocrine sweat glands limited to their paws. This sign may be absent or, if present, may be identified by moist impressions on the floor or ground where the canine walks.

#### Nicotinic Signs

Overstimulation of nicotinic receptors results in muscle fasciculation, weakness and paralysis. Paralysis of the phrenic nerve, which innervates the diaphragm, can lead to respiratory paralysis (inability to ventilate).

#### Confounders

Overstimulation of the nicotinic receptors at the autonomic ganglia can lead to stimulation of the parasympathetic and/or sympathetic nervous system. Stimulation of the parasympathetic system resembles muscarinic receptor overstimulation (see above). On the other hand, stimulation of the sympathetic nervous system can lead to hypertension, mydriasis, and tachycardia, confounding the identification of this toxidrome. In high dose exposures involvement of GABA and NMDA glutamate receptors may occur in the CNS. The heterogeneous nature of the clinical signs makes identification of this syndrome challenging.

A retrospective study in 102 dogs with OP or carbamate toxicosis reported that dogs presenting with classical muscarinic signs of intoxication (e.g., SLUDGE, triple B) had lower morbidity and mortality compared with dogs presenting with CNS signs (e.g., weakness, shock) (34). The authors suggested that earlier presentation (e.g., before CNS signs manifest) may be associated with a better outcome. Alternatively, identification of this toxidrome may be more likely when classic signs are apparent, leading to more rapid antidote administration.

#### Intermediate Syndrome

The intermediate form is more likely to be seen with longer term exposure. It may be seen when a canine is exposed to lipophilic organophosphates and results in a delayed onset of decreased appetite, depression, weakness, muscle tremors, abnormal behavior and ventroflexion of the neck (9).

#### Treatment

##### Standard Decon and PPE Required

If dealing with an unknown exposure, it may be prudent to follow FGA decon (see below). Atropine administration is used to control the muscarinic signs but not the nicotinic signs and, with known exposure, is the first line treatment. It is repeated until secretions are controlled and bradycardia improves. See Table 2 for dosages.

In addition to atropine, oximes (e.g., 2-PAM®) are administered for muscarinic, nicotinic and CNS signs (10, 11). If presented with an unknown toxicosis with clinical signs suggestive of a cholinergic agent a test dose of atropine at 0.02 mg/kg (typical anesthetic dosage) should be considered. If the eyes are dilated, and the heart rate increased and/or salivation stops within 10–15 min the syndrome is unlikely to be due to organophosphate or carbamate toxicosis. If the clinical signs do not improve the higher dose of atropine should be considered. The higher dose of atropine given to a canine that does not have cholinergic toxicosis will cause signs of atropine toxicosis (behavior changes, hyperthermia, erythema, dry mouth +/- seizures), which would need to be treated symptomatically.

Pralidoxime chloride (2-PAM® Protopam®) is in a class of chemicals (oximes) that reverse binding of cholinesterase inhibitors bound to acetylcholinesterase. It binds to the inhibitor, removing it from the enzyme, in a process called regeneration. It is effective before the toxin has irreversibly bound to the receptor (called aging) but not afterwards and aging varies with different agents. Some OP toxins age rapidly (e.g., minutes) and some take over 40 h. Pralidoxime was thought to be ineffective after 48 h (due to aging), however some toxins in this class are stored in fat and released over days to weeks and in these cases pralidoxime chloride would be effective. Pralidoxime is used for treatment of organophosphate toxicosis, however its use for carbamate toxicosis is controversial. Because carbamates usually bond for only ~1–2 h it was assumed the use of 2-PAM would not be effective by the time it was given. Additionally, one study showed that, when used alone (e.g., without atropine), for one specific carbamate toxin (e.g., carbaryl) the clinical signs worsened (35). When 2-PAM was given along with atropine, however, it worked successfully (36). Recent studies suggest that a higher dosage than originally published may be required (37). However, there are no clinical studies in dogs. Studies in rabbits demonstrated that 2-PAM in combination with atropine, resulted in a 35-fold increase in efficacy than administration of atropine alone (38). Due to the likelihood of mixed toxins, the low rate of side effects, and the improved effectiveness of atropine, 2-PAM should be used along with atropine for all organophosphate and carbamate toxicoses showing clinical signs.

Supportive care includes maintaining the airway and supporting ventilation, seizure control (e.g., treatment with diazepam or midazolam), tremor control to avoid worsening hyperthermia (e.g., methocarbamol), and decontamination (discussed below under FGAs). Drugs to avoid include phenothiazines (e.g., acepromazine), opioids, theophylline, and aminoglycosides.

*Clinical Pearl: Classic signs in dogs are excess secretions and muscle tremors or twitching. These may be accompanied by parasympathetic signs (miosis, bradycardia) or sympathetic signs (mydriasis, tachycardia). A classic sign for humans, profuse sweating, does not occur in dogs*.

### Fourth Generation Nerve Agents (FGAs)

Fourth generation nerve agents, also called Novichoks or A-series nerve agents are unique high potency organophosphate compounds with delayed and persistent activity. Novichoks are the binary form of the toxin with the final agent being labeled with a code number (e.g., A-234). Binary agents have two components that form the final toxin when combined. They may be dispersed in an ultrafine powder or can be liquid or aerosol. There are ~10,000 lethal doses in one gram of A-230.

#### Clinical Signs

Onset of clinical signs depends on the agent and route of exposure with rapid onset for inhalation and delated onset after dermal exposure. Classic clinical signs associated with lower generation nerve agents occur with FGAs as well and include salivation, lacrimation (tearing and nasal secretions), miosis, bronchorrhea (bronchial secretions), labored breathing and respiratory failure. In animal studies using FGAs bronchoconstriction and seizure activity occurred (39). It is unclear if these will be prominent clinical features in future exposures of humans or canines. Muscle tremors, twitching, confusion and ataxia can occur. Exposure may be difficult to distinguish from opioids if collapse has occurred and naloxone should be considered if exposure is unknown.

#### Treatment

##### FGA Decon (See Below) and PPE Required

Decontamination is done carefully as FGAs persist for a long time in water (e.g., water waste becomes hazardous) and on surfaces, possibly for several months. Patient decontamination, as described under PPE and decontamination, is started with the 10 s dry blot first (e.g., blot the canine's skin using a dry paper towel, paying particular attention to the hairless areas (e.g., groin, under front limbs) (see decon section for remainder). All water waste is to be considered contaminated. Avoidance of hand sanitizer or any alcohol based cleaning agent is recommended as these may enhance absorption of FGAs.

Treatment is centered around anticholinergics (e.g., atropine), oxime AChE reactivators (e.g., pralidoxime chloride; 2-PAM), and anti-seizure medications (diazepam, midazolam). Treatment is similar to nerve agent toxicosis (above) although much higher dosages are required with FGAs. FGAs are as or more potent and significantly more persistent than previous generation nerve agents. They require aggressive and sustained supportive care, higher amounts of antidotes and repeat dosages (39). The higher dosages of atropine are needed to treat the effects of FGAs but can lead to severe side effects. Originally, 2-PAM was not thought to be effective for FGAs, however clinicians treating patients in the 2018 attacks found it to be helpful in maintaining blood pressure and renal perfusion for unknown reasons (39). Atropine is given to effect (e.g., airway secretions drying and improvement of labored breathing) and 2-PAM is given until nicotinic effects diminish (e.g., muscle weakness). In severe toxicoses, anti-seizure medication is recommended even in the absence of clinical seizures. This is due to synergism of these medications with other drugs used that may potentiate the reversal. Seizures may be non-responsive to common anticonvulsants (e.g., levetiracetam, phenytoin). Medications can be given by auto-injector (IM) if available or via the intravenous route (preferred).

There are several AI (autoinjector) products that are pre-packaged for nerve agent poisoning including DuoDote® (atropine and 2-PAM in same Auto-injector), Antidote Treatment Nerve Agent Autoinjector (ATNAA; atropine and 2-PAM in same Autoinjector), and the Mark I kit (atropine and 2-PAM as separate AIs included in one package). The currently available AIs contain 2 mg of atropine and 600 mg of 2-PAM. Additionally, atropine comes in dosages of 2.0, 1.0, 0.5, and 0.25 mg and 2-PAM comes in 600 mg dosages and can be purchased separately.

With severe bronchoconstriction, not improved with atropine therapy, albuterol via delivery chamber should be considered at one puff up to q12h (14). Mechanical ventilation may be required so transport to a veterinary emergency facility with ventilator capabilities is essential.

Intravenous lipid emulsion (ILE) therapy may be an effective antidote. Many organophosphates are highly lipid soluble and in a meta-analysis of 7 randomized clinical trials with 630 patients, lipid therapy was associated with decreased morbidity and mortality. Lipids were associated with higher levels of complete cure (OR 2.54), lowered levels of mortality (OR 0.31), lowered liver enzymes and increased AChE (40). In the majority of these studies lipids were used at the standard dosage of 1.5 mL/kg of 20% lipid emulsion given IV. This is followed by 0.25 to 0.5 mL/kg/min as a constant rate infusion for 30–60 min. With increased accessibility, a growing body of literature, and a good safety profile, it is likely that ILE therapy may become a first line therapy for FGAs in the future.

Additional treatments used in humans are magnesium sulfate (bronchodilator, vasodilator) and corticosteroids (1–2 mg/kg methylprednisolone). Additionally, the U.S. Army found that galantamine at 5–8 mg/kg was synergistic with atropine, allowing a lower dose of atropine to be effective, thereby avoiding many of the side effects of high dose atropine. This drug may become an option in the future (39).

*Clinical Pearl: Fourth generation nerve agents are extremely toxic and consideration should be given to treatment with IV lipids. See* *Table 2* *for dosages*.

### Asphyxiants/Knockdown Agents

#### Overview

This category is separated into simple and central/chemical asphyxiants. Nitrogen and methane are examples from the simple category and these work by physically displacing oxygen from inspired air. The central asphyxiants either interfere with oxygen transport (e.g., carbon monoxide), or interfere with oxidative phosphorylation (e.g., hydrogen sulfide, cyanide, phosphine, azides) or both (carbon monoxide) (41). All lead to tissue hypoxia resulting in anaerobic metabolism and lactic acidosis.

#### Clinical Signs

With severe exposure, the dog may suddenly collapse and develop cardiac arrest. Severe toxicoses may cause seizures, coma, bradypnea or apnea, bradycardia and hypotension. Milder or early cases include restlessness, fatigue, tachypnea, tachycardia, and vomiting (41). Most will have lactic acidosis and when lactate is very high (>10 mmol/L) after smoke inhalation it is thought to indicate cyanide toxicosis in humans (42).

Carbon monoxide may result in a cherry red color to the mucous membranes and tachypnea along with normal tests of oxygenation (e.g., normal SpO_2_, due to pulse oximetry reading carbon monoxide as oxygen). There may be a rotten egg odor with hydrogen sulfide and, due to its caustic effects, corneal ulcerations may be seen. With cyanide toxicosis there may be an almond smell to the breath and/or a cherry red coloration of the skin, which is best seen in the relatively hairless groin are) (17). If lactate testing is available it may help discriminate mild to severe toxicosis. Normal lactate in canines is <2 mmol/L. There are no reported cut off values to identify severity in canines with asphyxiant toxicosis.

#### Treatment

##### Standard Decon and PPE Required

Immediate removal to fresh air, followed by administration of oxygen are recommended for all. For carbon monoxide toxicosis administration of oxygen should be provided as soon as possible. A canine specific oxygen mask is ideal, as the flatter masks for humans are hard to fit over the long-nosed canine. Oxygen appears to be beneficial with cyanide and hydrogen sulfide toxicosis despite the cytochrome oxidase inhibition (43). If cyanide or hydrogen sulfide are suspected hydroxycobalamin is the preferred treatment and is used at a much higher dosage than used for Vitamin B12 deficiency. The hydroxycobalamin exchanges a hydroxyl group for cyanide and the non-toxic product, cyanocobalamin, is excreted in the urine. In the absence of cyanide hydroxycobalamin binds to nitric oxide, which can be helpful if the animal is in circulatory shock (e.g., severe hypotension). Since each hydroxycobalamin molecule can only bind one cyanide ion, due to molecular weight differences, 52 grams of hydroxycobalamin are needed to bind 1 gram of cyanide. A study in adult Beagle dogs reported 100% survival in dogs given a lethal dose of cyanide that were treated with 150 mg/kg of hydroxycobalamin intravenously over 7.5 min. There was a lower survival in dogs given lower dosages of hydroxycobalamin (15). For reference, the dosage for human adults is 5 grams and the dosage recommended for vitamin B12 deficiency in dogs is 600 mcg/9–18kg K9 (vetmed.tamu.edu/gilab/research/cobalamin-information/). For cyanide toxicosis, the K9 dose would be 3.75 grams (e.g., 150 mg/kg for 25 kg K9) IV over 7.5 min. Side effects of this dosage of hydroxycobalamin in humans include red skin, red urine, a skin rash, hypertension, and, in children given a high dose, methemoglobinemia. Side effects in dogs are unknown.

If hydroxycobalamin is unavailable, thiosulfate or nitrite plus thiosulfate are options (17). Nitrite forms methemoglobin which then removes cyanide from cytochrome oxidase. Thiosulfate converts cyanide to thiocyanate, a less toxic alternative. If possible, transporting the canine to a facility with hyperbaric oxygen availability may be helpful.

*Clinical Pearl: Removal to fresh air for all. Oxygen therapy for all. Hydroxycobalamin for cyanide and hydrogen sulfide*.

### Solvents/Anesthetics/Sedatives (SAS)

#### Overview

This is a heterogeneous category with variations in clinical signs according to the specific agent. There are solvents (gasoline, benzene, toluene, xylene, carbon tetrachloride, freon), anesthetics (phenobarbital, pentobarbital, chloroform, halothane, nitrous oxide) and sedatives (diazepam, midazolam, alprazolam). These agents affect the central nervous and/or peripheral nervous system and may have secondary cardiac effects. Additionally, skin, gastrointestinal, hepatic, renal, and coagulation systems may be affected.

#### Clinical Signs

Early signs may include sedation, agitation (rare) or ataxia and mildly dull mentation or other behavioral changes. Nystagmus and chemical dermatitis may be seen as well as seizures and cardiac arrhythmias. CNS depression may progress to stupor and coma. Decreased respiratory rate may progress to respiratory and cardiac arrest. If exposure leads to high inhaled doses of solvents the first symptom may be sudden cardiac arrest (44, 45).

#### Treatment

##### Standard Decon and PPE Required

Removal from exposure is followed by control of the airway in dogs with severe clinical signs. Endotracheal intubation followed by ventilation may be lifesaving until arrival at a veterinary hospital.

Endotracheal Tube sizes are listed in Table 1 (See section Opioids/Opiates Opioid Toxicosis Treatment for details).

*Clinical Pearl: For unknown sedatives or those with no reversal agent consider naloxone. If the dog is not ventilating secure the airway and begin ventilations on the way to veterinarian*.

### Primary Respiratory Agents

#### Overview

Respiratory agents can affect the upper airway, the lower airway, or both. The damage caused by these agents is due to their alkaline nature (e.g., ammonia) or their acidic nature (e.g., chlorine, phosgene, nitrogen, etc.), as well as to the reactive oxygen species formation and inflammation that ensue (46). Clinical signs may be seen immediately or may be delayed.

Highly water-soluble (e.g., ammonia) and moderately water soluble (e.g., chlorine) chemicals affect the upper airway immediately and lead to rapid onset of mouth, throat, and eye irritation. These can progress to upper airway obstruction and subsequent cardiopulmonary arrest (47, 48).

Water insoluble compounds lead to damage to the lower airways and the onset may be delayed for hours to days. Damage to the lungs leads to acute lung injury which can progress to acute respiratory distress syndrome. Examples include phosgene and nitrogen oxide. Nitrogen oxides cause silo filler's disease, lung damage from inhalation of nitrogen oxides present in fresh silage.

#### Clinical Signs

The immediate nature of the clinical signs associated with water-soluble compounds may help limit exposure. Irritation of the eyes, nose and mouth occur rapidly and progress to upper airway signs that may include loud inspiratory noise (stertor or stridor), hoarse barking, coughing, and tachypnea. Complete upper airway obstruction may necessitate emergency airway access (14, 48).

Water insoluble compound exposure causes delayed clinical signs (hours to days after exposure) that may be difficult to connect to the event. Labored breathing, tachypnea, and cough are common and wheezes may be auscultated. Thoracic radiographs may show pulmonary edema. Sequelae to exposure include hyper-reactive airways (frequent bronchoconstriction to seemingly innocuous particulate matter), bronchiectasis and pulmonary fibrosis.

#### Treatment

##### Standard Decon and PPE Required

Water soluble compound exposure is treated with removal from the exposure, oxygen supplementation, flushing of the eyes with physiologic saline, and, if needed, bronchodilators (albuterol inhaler attached to delivery chamber such as Aerodawg®). Parenteral glucocorticoids may be helpful although this is unproven. Nebulization of sodium bicarbonate is used for chlorine inhalation (16).

Water insoluble compound exposure is treated with N-acetylcysteine, bronchodilators (albuterol inhalers attached to delivery chamber) and possibly, corticosteroids. Corticosteroid treatment has not been proven to affect outcome. Oxygen supplementation is NOT used for known phosgene exposure until absolutely necessary as oxygen induced lung injury may occur with oxygen supplementation after exposure to phosgene (49).

*Clinical Pearl: Oxygen for all except known phosgene exposure. Flush eyes and mouth and consider bronchodilators (e.g., albuterol via chamber)*.

### Vesicant/Riot Control/T2 Toxin

Vesicants are irritant, caustic chemicals that cause respiratory symptoms along with irritation of the skin and eyes. Examples of vesicants are nitrogen, phosgene, sulfur mustard, nitrogen mustard, and lewisite. Mustards are alkylating agents and affect eyes, skin, mucous membranes on contact by direct DNA damage. Mustards also increase oxidative stress and inflammation and result in depletion of endogenous anti-oxidants, particularly glutathione (47, 48).

#### Clinical Signs

Signs may be delayed for up to 24 h after exposure. Ocular injury includes red, irritated conjunctiva, blepharospasm, lid edema, and corneal ulcers. Skin injury begins as dermal separation from the epidermis. Inflammation, erythema, and pain are hallmarks. Vesicles coalesce into blisters, a distinguishing feature of this toxidrome. These are best visualized in the hairless regions (e.g., groin, under forelimbs). Vesicants are differentiated from large airway toxin exposure by the presence of skin blisters. Penetration of vapor may settle in inguinal and axillary areas so it is imperative to check these areas. Inhalation of mustard vapor leads to inflammation of the airways, acute respiratory distress syndrome, burning in the throat, and epistaxis. Later onset systemic effects include bone marrow suppression (neutropenia), sepsis, and potentially carcinogenesis (47, 48).

#### Treatment

##### Standard Decon and PPE Required

Ophthalmic ointments may provide some relief and prevent further damage. If inhalation has occurred providing oxygen (via canine specific oxygen mask) as well as transport to a veterinary emergency facility are essential. Other considerations if inhalation has occurred are administration of N-acetylcysteine, anticoagulants, and anti-inflammatory agents (hydrocortisone). See Table 2 for dosages.

*Clinical Pearl: Blisters are a classic sign, particularly in the groin and other hairless regions. Oxygen during transport to veterinarian essential except for known phosgene exposure*.

### Anticholinergic Toxicosis

Anticholinergic agents act by inhibiting the muscarinic receptors, mainly associated with the parasympathetic nervous system. The sympathetic nervous system innervates sweat glands, which are regulated by muscarinic receptors as well. The result is under-stimulation of cholinergic receptors. Examples include BZ (3-quinuclidinyl benzilate), and other anticholinergics (e.g., atropine, hyoscyamine, scopolamine). Exposure is via ingestion, inhalation, or dermal absorption.

#### Clinical Signs

Sayings such as “Blind as a bat,” “dry as a bone,” “hot as hades,” “red as a beet,” “mad as a hatter,” “full as a flask” (full urinary bladder) can help to remember the clinical signs, some of which are specific to humans (50). Early urinary catheter placement may alleviate the discomfort associated with urinary retention. Humans have specific hallucinations (e.g., Lilliputian) and actions (wool gathering) that have not been described in dogs. Also, signs such as anhidrosis (lack of sweating), and flushing are either absent, minimal or difficult to detect in canines. The canine hair coat covers up reddened or dry skin. Tachycardia and hypertension (systolic and diastolic) may occur (51).

Anticholinergic toxicosis results in mental dullness or delirium, mydriasis, dry mouth, tachycardia, hyperthermia, and decreased sweating (humans). Since dogs only sweat from their footpads and use evaporation (from panting) to cool themselves this sign is not apparent in canine toxicosis.

#### Treatment

##### Standard Decon and PPE Required

Supportive care consists of cooling and administration of benzodiazepines (e.g., diazepam, midazolam) for agitation or seizures, and IV fluids. Physostigmine or neostigmine should be considered as options. Physostigmine is given at 1 mg IV per adult human. Neostigmine, an anticholinesterase, allows acetylcholine to persist and is given at dose of 0.01 to 0.02 mg/kg SC or IM. If mental dullness progresses consider naloxone. See Table 2 for dosages.

*Clinical Pearl: The signs of this toxidrome are difficult to distinguish in dogs. Mental dullness and tachycardia are seen with many different conditions. If exposure is unclear consider naloxone*.

### Unknown Toxicosis

A thorough review of identification and management of the unknown toxicant has been published (6). When there is a strong clinical suspicion that a working canine has been exposed to an unknown toxin and the clinical signs include sedation, mental depression or respiratory depression, a trial dose of naloxone should be considered. If the clinical signs fail to respond or worsen after repeated doses of naloxone and the canine is deteriorating, IV lipid therapy should be considered. See Table 2 for dosages.

## Conclusion

Identification of toxidromes with canine specific clinical signs as well as antidote dosages and supportive care are likely to become an essential tool for veterinarians and first responders. As their global value is recognized, working dogs are being utilized more frequently and in more varied scenarios. With the increase in working dogs in the field it is imperative that we provide them with a similar level of protection as their human partners.

## Data Availability Statement

The original contributions presented in the study are included in the article/supplementary material, further inquiries can be directed to the corresponding author/s.

## Author Contributions

MM provided substantial contributions to the conception and design of the work, drafted the initial work, provided critical revisions, provided approval for publication, and agrees to be accountable for all aspects of the work in ensuring that questions related to the accuracy or integrity of any part of the work are appropriately investigated and resolved. MS and BA provided substantial contributions to the conception and design of the work, provided critical revisions, provided approval for publication, and agree to be accountable for all aspects of the work in ensuring that questions related to the accuracy or integrity of any part of the work are appropriately investigated and resolved. All authors contributed to the article and approved the submitted version.

## Conflict of Interest

The authors declare that the research was conducted in the absence of any commercial or financial relationships that could be construed as a potential conflict of interest.

## Publisher's Note

All claims expressed in this article are solely those of the authors and do not necessarily represent those of their affiliated organizations, or those of the publisher, the editors and the reviewers. Any product that may be evaluated in this article, or claim that may be made by its manufacturer, is not guaranteed or endorsed by the publisher.

## References

[1] Holstege CP Borek HA. Toxidromes. Crit Care Clin. (2012) 28:479–98. 10.1016/j.ccc.2012.07.008 22998986

[2] Ciottone GR. Toxidrome recognition in chemical weapons attacks. N Engl J Med. (2018) 378:1611–20. 10.1056/NEJMra1705224 29694809

[3] Gwaltney-Brant SM Murphy LA Wismer TA Albretsen JC. General toxicologic hazards and risks for search-and-rescue dogs responding to urban disasters. JAVMA. (2003) 222:292–5. 10.2460/javma.2003.222.292 12564589

[4] Murphy LA Gwaltney-Brant SM Albretsen JC Wismer TA. Toxicologic agents of concern for search-and-rescue dogs responding to urban disasters. JAVMA. (2003) 222:296–304. 10.2460/javma.2003.222.296 12564590

[5] Wismer TA Murphy LA Gwaltney-Brant SM Albretsen JC. Management and prevention of toxicosis in search-and-rescue dogs responding to urban disasters. JAVMA. (2003) 222:305–9. 10.2460/javma.2003.222.305 12564591

[6] Brutlag AG Hovda L Poppenga RH. Identification and management of the unknown toxicant. In: Hovda L Brutlag AG Poppenga RH Peterson KL, editors. Blackwell's Five-Minute Veterinary Consult Clinical Companion: Small Animal Toxicology, 2nd ed. Ames, IA: Wiley Blackwell (2016). pp. 49–59.

[7] Palmer L. Operational canines. Vet Clin North Am Small Anim Pract. (2021) 51:945–60. 10.1016/j.cvsm.2021.04.011 34059266

[8] Essler JL Smith PG Berger D Gregorio D Pennington MR McGuire A . A randomized cross-over trial comparing the effect of intramuscular versus intranasal naloxone reversal of intravenous fentanyl on odor detection in working dogs. Animals. (2019) 9:E385. 10.3390/ani9060385 31234512 PMC6617369

[9] Means C. Organophosphate and carbamate insecticides. In: Peterson ME Talcott PA, editors. Small Animal Toxicology. 3rd ed. St. Louis, MO: Saunders-Elsevier (2013). pp. 715–24.

[10] Boothe DM. Boothe's Small Animal Formulary. 7th ed. Lakewood, CO: American Animal Hospital Association Press (2015). p. 16. Atropine.

[11] Boothe DM. Boothe's Small Animal Formulary. 7th ed. Lakewood, CO: American Animal Hospital Association Press (2015). p. 148. Pralidoxime.

[12] Boothe DM. Boothe's Small Animal Formulary. 7th ed. Lakewood, CO: American Animal Hospital Association Press (2015). p. 119. Midazolam.

[13] Boothe DM. Boothe's Small Animal Formulary. 7th ed. Lakewood, CO: American Animal Hospital Association Press (2015). p. 49. Diazepam.

[14] Cote E. Drug formulary. In: Cote E, editor. Clincal Veterinary Advisor Dog and Cat. 3rd ed. St. Louis, MO: Elsevier (2015). p. 1511.

[15] Borron SW Stonerook M Reid F. Efficacy of hydroxycobalamin for the treatment of acute cyanide poisoning in adult Beagle dogs. Clin Toxicol. (2006) 44(Suppl. 1):5–15. 10.1080/15563650600811672 16990189

[16] Vajner JE III Lung D. Case files of the University of California San Francisco Medical Toxicology Fellowship: acute chlorine gas inhalation and the utility of nebulized sodium bicarbonate. J Med Toxicol. (2013) 9:259–65. 10.1007/s13181-013-0309-8 23719961 PMC3770993

[17] Fitzgerald KT. Cyanide. In: Peterson ME Talcott PA, editors. Small Animal Toxicology. 3rd ed. St. Louis, MO: Saunders-Elsevier (2013). pp 523–7.

[18] Boothe DM. Boothe's Small Animal Formulary. 7th ed. Lakewood, CO: American Animal Hospital Association Press (2015). p. 144. Physostigmine.

[19] Boothe DM. Boothe's Small Animal Formulary. 7th ed. Lakewood, CO: American Animal Hospital Association Press (2015). p. 126. Neostigmine.

[20] Venable E Discepolo D Powell E Liang SY. An evaluation of current working canine decontamination procedures and methods for improvement. J Vet Behav. (2017) 21:53–58. 10.1016/j.jveb.2017.07.008 29104518 PMC5665018

[21] Murphy LA. Responding to mass exposures. In: Peterson ME Talcott PA, editors. Small Animal Toxicology, 3rd ed. St. Louis, MO: Saunders-Elsevier (2013). pp. 159–65.

[22] Wismer T. Induction of vomiting. In: Cohn LA Cote E, editors. Clinical Veterinary Advisor Dogs and Cats. 4th ed. St. Louis, MO: Elsevier (2020). p. 1188–89.

[23] Suokko M Saloranta L Lamminen T Laine T Elliott J. Ropinirole eye drops induce vomiting effectively in dogs: a randomized, double-blind, placebo controlled clinical study. Vet Rec. (2019) 186:283. 10.1136/vr.104953 31409749 PMC7063390

[24] *Broward County K9 Opioid Exposure*. Available online at: https://workingdoghq.com/the-dangers-of-opioids-for-working-dogs/ (accessed January 03, 2022).

[25] Chodoff P Domino EF. Comparative pharmacology of drugs used in neuroleptanalgesia. Anesth Analg. (1965) 44:558–63. 10.1213/00000539-196509000-00018 5897019

[26] Janssen PA. Potent, new analgesics, tailor made for different purposes. Acta Anaesthesiol Scand. (1982) 26:262–8. 10.1111/j.1399-6576.1982.tb01765.x 7113634

[27] Alinejad S Kazemi T Zamani N Hoffman RS Mehrpour O. A systematic review of the cardiotoxicity of methadone. EXCLI J. (2015) 14:577–600. 10.17179/excli2015-553 26869865 PMC4747000

[28] Roan JP Bajaj N Davis FA Kandinata N. Opioids and chest wall rigidity during mechanical ventilation. Ann Intern Med. (2018) 168:678. 10.7326/L17-0612 29310135

[29] Soares JHN Henao-Guerrero N Pavlisko ND Williamson A Giannellge-neto A. The effect of two doses of fentanyl on chest wall rigidity at equipotent doses of isoflurane in dogs. Vet Anaesth Analg. (2019) 46:360–4. 10.1016/j.vaa.2019.01.001 30772260

[30] Kleine S Quandt J. Opiates and opioids. In: Hovda L Brutlag AG Poppenga RH Peterson KL, editors. Blackwell's Five-Minute Veterinary Consult Clinical Companion: Small Animal Toxicology. 2nd ed. Ames, IA: Wiley Blackwell (2016). pp 217–26.

[31] Mitek AE McMichael MA Weir WB Smith MJ Schneider DC. The Carle-Illinois treatment protocol for law enforcement K9s: guidelines for emergency medical services. Prehosp Disaster Med. (2019) 34:428–37. 10.1017/S1049023X19004448 31244452

[32] Talcott P. Organophosphate and carbamate insecticides. In: Hovda L Brutlag AG Poppenga RH Peterson KL, editors. Blackwell's Five-Minute Veterinary Consult Clinical Companion: Small Animal Toxicology. 2nd ed. Ames, IA: Wiley Blackwell (2016). pp. 689–95.

[33] Gupta RC Milatovic D. Organophosphates and carbamates. In: Gupta RC, editor. Veterinary Toxicology: Basic and Clinical Principals. 2nd ed. London: Saunders-Elsevier (2012). pp. 573–85.

[34] Klainbart S Grabernik M Kelmer E Chai O Cuneah O Segev G . Clinical manifestations, laboratory findings, treatment and outcome of acute organophosphate or carbamate intoxication in 102 dogs: a retrospective study. Vet J. (2019) 251:105349. 10.1016/j.tvjl.2019.105349 31492383

[35] Harris LW Talbot BG Lennox WJ Anderson DR. The relationship between oxime-induced reactivation of carbamylated acetylcholinesterase and antidotal efficacy against carbamate intoxication. Toxicol Appl Pharmacol. (1989) 98:128–33. 10.1016/0041-008X(89)90140-3 2494778

[36] Natoff IL Reiff B. Effect of oximes on the acute toxicity of anticholinesterase carbamates. Toxicol Appl Pharmacol. (1973) 25:569–75. 10.1016/0041-008X(73)90026-4 4741778

[37] Howland MA Aaron C. Antidotes in depth: pralidoxime. In: Goldfrank LR Weisman RS Flomenbaum NE Howland MA Lewin NA Hoffman RS, editors. Goldfrank's Toxicologic Emergencies. Norwalk, CT: Appleton & Lange (1994). pp. 1117–19.

[38] O'Leary JF Kunkel AM Jones AH. Efficacy and limitations of oxime-atropine treatment of organophosphorus anticholinesterase poisoning. J Pharmacol Exp Ther. (1961). 132:50–57. 13730087

[39] CHEMM Chemical Hazards Emergency Medical Management U.S. Department of Health and Human Services. Fourth Generation Agents. Downloaded January 15, 2020. Available online at: https://chemm.nlm.nih.gov/nerveagents/FGA.htm.

[40] Yu S Yu S Zhang L Gao Y Walline J Lu X . Efficacy and outcomes of lipid resuscitation on organophosphate poisoning: a systematic review and meta-analysis. Am J of Emerg Med. (2019) 37:1611–17. 10.1016/j.ajem.2018.11.022 30527914

[41] Fitzgerald KT. Carbon monoxide. In: Peterson ME Talcott PA, editors. Small Animal Toxicology. 3rd ed. St. Louis, MO: Saunders-Elsevier (2013). pp. 479–87.

[42] Baud FJ Barriot P Toffis V Riou B Vicaut E Lecarpentier Y . Elevated blood cyanide concentrations in victims of smoke inhalation. N Eng J Med. (1991) 325:1761–6. 10.1056/NEJM199112193252502 1944484

[43] Borron SW Bebarta VS. Asphyxiants. Emerg Med Clin North Am. (2015) 33:89–115. 10.1016/j.emc.2014.09.014 25455664

[44] Plumb D. Diazepam. In: Plumb's Veterinary Drug Handbook. 8th ed. Ames, IA: Wiley-Blackwell (2015). p. 424.

[45] Plumb D. Phenobarbitol Overdose, in Plumb's Veterinary Drug Handbook. 8th ed. Ames, IA: Wiley-Blackwell (2015). p. 1145.

[46] Knight MW. Zinc phosphide. In: Peterson ME Talcott PA, editors. Small Animal Toxicology. 3rd ed. St. Louis, MO: Saunders-Elsevier (2013). pp. 853–63.

[47] Fitzgerald KT. Smoke inhalation. In: Peterson ME Talcott PA, editors. Small Animal Toxicology. 3rd ed. St. Louis, MO: Saunders-Elsevier (2013). pp. 409–22.

[48] Sullivan JB Van Ert MD Krieger GR Peterson ME. Indoor environmental quality and health. In: Peterson ME Talcott PA, editors. Small Animal Toxicology. 3rd ed. St. Louis, MO: Saunders-Elsevier (2013). pp.139–58.

[49] Hardison LS jr Wright E Pizon AF. Phosgene exposure: a case of accidental industrial exposure. J Med Toxicol. (2014) 10:51–6. 10.1007/s13181-013-0319-6 23842907 PMC3951639

[50] CHEMM Chemical Hazards Emergency Medical Management U.S. Department of Health and Human Services. Anticholinergic. Downloaded January 15. 2020. Available online at: https://chemm.nlm.nih.gov/Anticholinergic.htm.

[51] Plumb D. Atropine, in Plumb's Veterinary Drug Handbook. 8th ed. Ames, IA: Wiley-Blackwell (2015). p. 127–98.

